# Reading Comprehension and Metalinguistic Knowledge in Chinese Readers: A Meta-Analysis

**DOI:** 10.3389/fpsyg.2019.03037

**Published:** 2020-02-05

**Authors:** Yang Dong, Shu-Na Peng, Yuan-Ke Sun, Sammy Xiao-Ying Wu, Wei-Sha Wang

**Affiliations:** ^1^Faculty of Education and Science, Jiaying University, Meizhou, China; ^2^Department of Social and Behavioural Sciences, City University of Hong Kong, Kowloon Tong, Hong Kong; ^3^Department of Science and Environmental Studies, The Education University of Hong Kong, Tai Po, Hong Kong; ^4^Department of Special Education and Counselling, The Education University of Hong Kong, Tai Po, Hong Kong; ^5^University of Southampton, Southampton, United Kingdom

**Keywords:** reading comprehension, metalinguistic knowledge, phonological awareness, morphological awareness, meta-analysis

## Abstract

Metalinguistic knowledge has a facilitative effect on reading comprehension. This meta-analysis examined the relationship between metalinguistic knowledge and reading comprehension among Chinese students. By focusing on both Chinese and English scripts' reading comprehension performance, this study synthesized 46 studies with 73 independent samples that represented 10,793 Chinese students from primary school to university levels. We found that in both Chinese and English scripts' reading, morphological awareness had the strongest correlation with reading comprehension, whereas both phonological awareness and orthographical skill had a similar medium correlation with reading comprehension. All three metalinguistic knowledge, which was not significantly influenced by the selected moderators of grade group, area, language type, and assessment, had an independent correlation with reading comprehension. The results suggested that reading stages did not significantly impact the function of metalinguistic knowledge on both Chinese and English scripts' reading comprehension for Chinese students. In addition, for Chinese students, morphological awareness plays a more important role than phonological awareness and orthographical skill in both Chinese and English scripts' reading comprehension.

## Introduction

Reading comprehension refers to the text mental image construction that comes from the interaction between text content and readers' cognition process through vocabulary knowledge, inference ability, word reading, working memory, and background knowledge (Snow, 2002; LervAag et al., 2018). Reading comprehension is the foundation for knowledge acquisition, cultural engagement, and success in workplaces (Chik et al., 2012a; García and Cain, 2014; Cheng et al., 2017). When engaging in text comprehension activities, readers need more complex cognitive capacity and resources to achieve reading comprehension tasks. Metalinguistic knowledge, also known as “metalinguistic awareness” or “metalinguistic ability” about language, in reading comprehension directly worked on a single-word or single-character cognition process. Metalinguistic knowledge refers to the ability to consciously reflect on the nature of language as “knowing about knowing” on words' or characters' semantic meaning and structure organization identification (Alderson et al., 1997; Elder et al., 1999). Regarding the single-word/single-character identification process, past studies mainly investigated the metalinguistic knowledge effect through three perspectives: morphological awareness (MA), orthographical skill (OS), and phonological awareness (PA). Metalinguistic knowledge was regarded as the most powerful tool in basic reading ability measurement, which could be used to predict reading comprehension performance among school-aged students through word reading (reading fluency and reading accuracy) and relevant decoding ability such as the single-word identification (Hoover and Gough, 1990; Tighe et al., 2019). Recently, Castles et al. (2018) revealed that the foundation skill of metalinguistic knowledge might be PA for alphabetical language. However, Chinese logographic scripts' reading material has a very different cognition system from alphabetical scripts' reading material in terms of grammatical knowledge, character structure, and pronunciation rules (Tsai et al., 2012; Ehrich et al., 2013). Alphabetical language (e.g., English) followed spelling-to-sound mappings (Zhu et al., 2014); that is, most alphabetical languages have a left-to-right structure of letter strings and use letters mapping on to the phonemes. By contrast, logographic languages (e.g., Chinese) have less information on phonology; the logographic language construction followed the rule of graphic unit of the character rather than phoneme owing to the richness of homophones in Chinese (Kuo and Anderson, 2006). Past studies reported that a Chinese character syllable represents three to five morphemes (Chow et al., 2008). Whether (Castles et al., 2018) conclusion can be adapted to comprehension texts in logographic scripts, such as Chinese comprehension text, required further investigation. Moreover, little research has been conducted on the meta-analytic method to investigate the correlation between metalinguistic knowledge and reading comprehension in Chinese students whose background was logographical scripts. In addition, reading stage theory suggested that students started learning to read at early primary school and started reading to learn when the basic knowledge of reading was obtained; with reading experience increasing, students become more professional in reading to learn at the university level (Chall, 1983). Whether the effects of three metalinguistic knowledge on reading comprehension were the same for Chinese students in different reading stages required further investigation. To fill this gap, this study tries to investigate the correlation between three types of metalinguistic knowledge (PA, OS, and MA) and Chinese and English scripts' reading comprehension in Chinese students.

## Literature Review

### Metalinguistic Knowledge and Reading Comprehension

Metalinguistic knowledge was regarded as a prominent feature in cognitive factors to predict reading comprehension performance through decoding and word identification on the single-word or single-character cognition process (Hoover and Gough, 1990; Tighe and Schatschneider, 2016). Metalinguistic knowledge, which starts to operate when a child enters the language world, is regarded as meta-cognitive resources for new words or character comprehension (Castles et al., 2018). The current study mainly investigated the effect of MA, OS, and PA, which were selected by extensive studies as metalinguistic knowledge in the reading field.

#### Morphological Awareness

MA is the ability to manipulate words' morphemes and the morphological structure (Kuo and Anderson, 2006). Because the majority of word readers encountered in printed school materials are multimorphemic and semantically complex, MA is important for both eastern and western countries' students to guess word meanings (Nagy et al., 2006; McBride-Chang et al., 2008). For example, a study showed that MA of grade 2 Canadian students significantly predicted their reading comprehension in later study periods, even after taking into account the influences of general intelligence and grade 2 reading comprehension ability (Deacon and Kirby, 2004). Shu and Anderson (1997) reported that Chinese students in Grades 3 and 5 could improve reading comprehension performance by improving their radical awareness of morphemes of characters. In both English and Chinese reading comprehension tasks, students who had better word's radical knowledge resulted in better performance in reading comprehension tasks (Jeon and Yamashita, 2014). MA can enhance word or character recognition rates, which are partially known, and thereby expand the range of recognizable words or characters. For example, a study reported Hong Kong students' Chinese MA predicted their Chinese reading comprehension and students' English MA predicted English reading comprehension (McBride-Chang et al., 2003). However, past studies showed inconsistent results in the association, which varies from moderate to high, between MA and reading comprehension (Shu et al., 2006; Zhang H., 2013).

#### Phonological Awareness

PA is defined as the ability to manipulate the sounds of spoken language (Blachman et al., 1999). PA is the reader's sensitive cognition of the segmentation of sound structures through onset, rhyme, phoneme, and coda. This is a type of metalinguistic awareness that develops in school-aged students (Ziegler and Goswami, 2005). PA is also a great predictor of children's reading performance in both Chinese and English reading comprehension tasks (Ruan et al., 2018; Tibi and Kirby, 2018). PA contributes to the comprehension of alphabetical writing systems in which target words had predictable graphemes symbolizing phonemes. It also contributes to reading comprehension through various ways of word reading (Engen and Høien, 2002; Nakamoto et al., 2007; Melby-Lervåg and Lervåg, 2011). Firstly, the blending skill, one ability of PA, aims to transform graphemes into recognizable words (McBride-Chang et al., 2006). Secondly, onset-rime segmentation and blending skills can help readers with analogy reading. Thirdly, readers need to adapt the rule of graphemes to phonemes in the word for memory requirement (Shu et al., 2008; Castles et al., 2018). To comprehend a text, readers utilize to recognize many text words or characters for effective word reading. For example, a study reported that PA determined the process of Chinese participants' reading comprehension (Chow et al., 2005). However, the correlation between PA and reading comprehension was not consistent. Past studies showed different levels of correlation between PA and reading comprehension in Chinese students, which is from low to moderate correlation (Yan et al., 2007; Chen and Chen, 2008; Chang, 2010).

#### Orthographical Skill

OS refers to an ability to manipulate the rule of sound–symbol of characters (Koda, 2005; Muroya et al., 2017) and a sophisticated knowledge to manipulate irregular or infrequent words' spellings (Jeon and Yamashita, 2014). Previous research tested the unique contribution of OS to reading comprehension (Nassaji and Geva, 1999; Kato, 2009). For example, Kato (2009) found that OS is a predictor of reading comprehension performance in high-ability university-level learners whose second language was English. Meanwhile, research revealed that readers' orthographic sensitivity, developed along with their reading comprehension ability, relied more on OS than PA on the print word process with increasing age (Koda, 2007; Kato, 2009). Regarding Chinese reading comprehension, for example, Ho and Bryant (1997) reported that primary school Chinese readers' OS levels predicted their reading comprehension performances. However, past results showed varied correlations ranging from low to high between OS and reading comprehension in Chinese participants (Cheung et al., 2007; Jia, 2015; Xia et al., 2017).

Given the fact that the correlation picture between three metalinguistic knowledge and reading comprehension was not clear for Chinese students, the meta-analytic method should be applied to address this problem.

### Potential Moderators

#### Grade Group

Reading stage theory suggested that the aim of reading was different, from primary school students to university students, from learning to read at early primary school grades and transmit to reading to learn in later higher grades' reading career (Chall, 1983). Previous studies reported that metalinguistic knowledge contributed to students' reading comprehension since primary school to higher education (McGee et al., 2002; Walley et al., 2003; Castles et al., 2018). In addition, past meta-analytic review of language learning showed that grade group was a significant moderator for reading comprehension ability through different stages of learning period (Schatschneider et al., 2004; Lervåg and Aukrust, 2010; Melby-Lervåg and Lervåg, 2014). Therefore, the grade group was selected as a potential moderator.

#### Language Type

The grammatical knowledge for Chinese scripts was different from English scripts in function word sequences (Cheng et al., 2017; Chen et al., 2018). Past studies showed that the differences between logographical scripts (e.g., Chinese) and alphabetical scripts (e.g., English) were phonology, syntax, and semantic structure (Connor and Connor, 1996; Tsai et al., 2012; Ehrich et al., 2013). For example, Chinese scripts had its unique character structure in which most characters consisted of a radical part for the semantic function of the character and the object character for pronunciation function (e.g., McBride-Chang et al., 2003). The differences between Chinese scripts and English scripts may result in different correlations between metalinguistic knowledge and reading comprehension.

#### Area

There are two reasons for selecting the area as a potential moderator. Firstly, the writing version system is different between mainland China (simplified scripts) and Hong Kong and Taiwan (traditional scripts). Gao and Kao (2002) reported that the simplified version has ~22.5% fewer strokes than the traditional version. The characters from traditional scripts are more complicated than those from simplified scripts. Regarding character recognition process, the construction of each character may be different between simplified scripts and traditional scripts, which may result in students having different performances on MA and OS. Secondly, students from mainland China and Taiwan speak Mandarin, whereas students from Hong Kong speak Cantonese. It should be noted that the pronunciation for each character is also different, where students may have different performances in the PA task. In mainland China, students are introduced to a Pinyin system, which uses English letters to represent individual phonemes; however, in Taiwan, students are introduced to the Zhuyin Fuhao system, which transcribes spoken pronunciation at the onset-rime level.

#### Assessment

A substantial difference of assessment, also called measurement, effects is found between standardized measures and researcher-developed measures (Pike, 1996; Koretz, 2002), which can provide more proximal measures on whether participants are applying the target reading practices that used in their research. For example, Pyle et al. (2017) reported that the effect size of comprehension score was larger in researcher-developed measures than standardized measures. Measurement presentation also impacted the results. For example, in PA assessment, the difficulty of tasks was different between visual presentation and auditory presentation owing to working memory load (Arrington et al., 2014; Friedman et al., 2017).

### Review on Past Relevant Meta-Analysis

Extensive previous studies reported the effect of metalinguistic knowledge and reading comprehension for participants whose first language was alphabetical language (Bus and Van IJzendoorn, 1999; Ehri et al., 2001a,b; Swanson et al., 2003; Mayberry et al., 2011; Melby-Lervåg and Lervåg, 2011, 2014; Jeon and Yamashita, 2014). For example, Mayberry's team (Mayberry et al., 2011) reported that PA had a low-to-moderate correlation with reading comprehension in deaf individuals. Ehri et al. (2001a) revealed that the effect size of PA on reading comprehension was moderate. Ehri et al. (2001a) found that the effect size of PA was larger in preschool than grade 1 of primary school. Moreover, Ehri et al. (2001a) showed that class size was not a significant moderator for the correlation between PA and reading comprehension. Swanson's research team (Swanson et al., 2003) showed that when the extent of the relationship between PA and reading comprehension was moderate, the importance of PA on reading comprehension might be overstated. The correlation between metalinguistic knowledge and reading comprehension was not consistent. Melby-Lervåg and Lervåg (2011) found the correlation between PA and reading comprehension was a range from low to moderate perspective from the cross-linguistic transfer. Melby-Lervåg and Lervåg (2014) reported that the correlation effect size between PA and reading comprehension was large in L2 condition. Jeon and Yamashita (2014) reported that the correlation between L2 reading comprehension and metalinguistic knowledge was low.

Only a few studies reported the correlation for participants whose first language background was logographical language (e.g., Chinese). Tan et al. (2005) synthesized the correlation of PA between brain reading mappings and found that a higher correlation in L2 alphabetical language for Chinese participants. Song et al. (2016) reported that the PA had medium correlation with L2 reading in Chinese students. Ruan et al. (2018) reported that the MA had stronger correlation with reading comprehension than PA with Chinese reading comprehension, but they did not solve the significant heterogeneity problem, which resulted in a challenged conclusion. In addition, past studies had two obvious limitations. Firstly, past studies synthesized the correlation between metalinguistic knowledge and L2 reading comprehension, and few studies have investigated the effect size of metalinguistic knowledge on Chinese (L1) reading comprehension. Secondly, past studies only reported single factor's (e.g., PA) effect size on reading comprehension, and a few studies investigated and compared the effect size of MA, PA, and OS on reading comprehension. That is, a few studies written in either English or Chinese have attempted an investigation of the correlation between each metalinguistic knowledge (MA, PA, and OS) and reading comprehension for Chinese students over the past few decades.

### Rationale for This Meta-Analysis

This study has outlined a range of potential moderators (grade group, language type, area, and assessment) that might influence the strength of the correlation between metalinguistic knowledge and reading comprehension. Firstly, it would be difficult to take into account all these selected moderators in a single study. For example, the interaction effect of grade group on the correlation between three selected metalinguistic knowledge and reading comprehension could not be calculated in one empirical study. The available solution here is to conduct a meta-analysis to investigate the interaction effect across all possible grade groups. Secondly, with the various correlations between selected metalinguistic knowledge and reading comprehension in past empirical studies, this meta-analytic approach enables us to integrate the various results through various samples and research designs.

### The Current Meta-Analysis

The current study expands the current literature to investigate the correlation between metalinguistic knowledge and reading comprehension in three ways. Firstly, it is the only review using a meta-analytic approach to explore the effect of metalinguistic knowledge on reading comprehension for primary school, secondary school, and university Chinese students. Secondly, the current study investigated the effect of metalinguistic knowledge on Chinese students' reading comprehension, whose L1 was a logographical language that is fundamentally different from the alphabetical language. In addition, the current study investigated the three popular metalinguistic knowledge (MA, OS, and PA) correlation with reading comprehension and further explored the reading stage's effect contribution between metalinguistic knowledge and reading comprehension in Chinese students.

## Methods

The official guidance of Preferred Reporting Items for Systematic Reviews and Meta-Analyses (*PRISMA*, Moher et al., 2010) was applied to the current method. PRISMA provided the detailed information for a meta-analysis, which included literature base, inclusion criteria, coding process, and meta-analytic procedure. PRISMA also provided the suggestions on data analysis, such as the risk of bias in individual studies.

### Literature Base

All possible materials were related to Chinese students' reading comprehension research. Publications, including dissertations, journal articles, and thesis, that were written in Chinese and English were all included in the database. This study searched English literature from PsycINFO, Education Resources Information Center (ERIC), and Pro-Quest and Chinese literature from the China National Knowledge Infrastructure (CNKI) database, which included all possible academic materials around China. Two groups of key words were used when searching for target studies. The first group related to metalinguistic knowledge: morphological awareness^*^, morpheme discrimination^*^, compound structure^*^, meaning selection^*^, radical identification^*^, radical meaning^*^, radical discrimination^*^, morpheme^*^, phonological awareness^*^, onset detection^*^, tone discrimination^*^, phoneme^*^, rhyme awareness^*^, blending^*^, orthographic^*^, orthographic awareness^*^, position awareness^*^, and phonological awareness^*^. The second group includes reading comprehension (sentence comprehension^*^, paragraph comprehension^*^, passage comprehension^*^, text comprehension^*^, reading comprehension^*^, reading ability^*^, comprehension ability^*^, and reading acquisition^*^). We selected all possible materials from between January 1, 1998, and March 31, 2019, which yielded 1,105 articles.

### Inclusion Criteria

Materials were discarded when they have any of the following features: (a) single case studies; (b) opinion or non-empirical articles; (c) participants were not Chinese students; (d) participants were diagnosed with serious special education needs (e.g., deaf or blind); (e) qualitative research design; (f) participants' grade group was not clear or grade group was not independent; (g) if the research reported participants' second language acquisition, the second language was not English; and (h) the first language of participants was not Chinese. In addition, we only included materials with the following required information: (a) materials provided the necessarily available correlation scores between reading comprehension and metalinguistic knowledge; (b) all possible indicators (e.g., sample size, *r, R*^2^, *t*-value, and *p*-value), which could be used to measure the correlation effect size Fisher's *z*; (c) all participants were primary school students, secondary school students, and college or university students; and (d) both metalinguistic knowledge and reading comprehension should be measured at the same time, because we wanted to report the current correlation. These inclusion criteria were applied to research with students rather than working professionals because García and Cain (2014) revealed that working professionals would not improve their reading competencies as quickly as students would. Because the majority of Chinese participants started doing formal reading comprehension tasks since grade 1, we removed those studies that reported the correlation before grade 1.

### Coding Process

Two independent coders coded materials through (a) year of publication, (b) first author's surname, (c) area (Mainland, Hong Kong, Taiwan, or other countries), (d) material resources (written in Chinese or English), (e) sample size, and (f) grade group. According to reading stage theory suggestion, from learning to learn to be professional in reading to learn, the current study divided students' grade group into five grade groups: *L* for grade 1 and grade 2 of primary school, *M* for grade 3 and grade 4 of primary school, *H* for grade 5 and grade 6 of primary school, *S* for secondary school, and *U* for college and university students; (g) language type (L1 or L2); and (h) the effect size of Fisher's *z*. Any unclear information would be confirmed by the articles' author(s). If the study provided the correlation indicator in both standardized test and researcher-developed test, we reported the indicator from the standardized test. If one study provided more than one available effect sizes between one type of metalinguistic knowledge and reading comprehension (e.g., MA-reading comprehension), we followed the cluster regression method to calculate the unique effect size of the study (Hedges et al., 2010; Tanner-Smith et al., 2016), ensuring that each study only provided one effect size for a meta-analysis. If one article provided the correlation through different metalinguistic knowledge, [for example, one study provided two correlations (MA-reading comprehension and PA-reading comprehension) at the same time], we treated this article as two independent studies. If one article provided the correlation between metalinguistic knowledge and reading comprehension through mixed-grade groups, the authors were contacted for clear details; we removed those articles in which the key indicators were not clear (e.g., grade group, language type, and metalinguistic knowledge type).

Two coders coded materials independently. The first-round results of coding similarity ranged between 77 and 100% across all selected materials. The differences came from the sampling area; through contacting authors, two coders got consensus results on the sampling area.

### Meta-Analytic Procedures

There were eight outliers, with correlations >2.5 standard deviations from the mean (García and Cain, 2014); specifically, all outliers came from the correlation studies between MA and reading comprehension, and this study removed two studies from Catherine (nos. 20a and 20b), two studies from Chen Jing (nos. 21 and 47), two studies from Katie (nos. 52a and 52b), and two studies from Zhang (2016) (nos. 15 and 38), resulting in a final number of 46 articles with 73 independent studies (total *N* = 10,793). This study followed the analytic procedures by Mol and Bus (2011). All correlation indicators were entered into comprehension meta-analysis to transform into Fisher's *z*. We selected Fisher's *z* as effect size because the variance of *z* is approximately constant, whereas the variance of the correlation follows an asymmetrical distribution (Borenstein et al., 2009). According to the Cohen (1988) interpretation on Fisher's *z*, a Fisher's *z* value of 0.10 (*r* = 0.10) can be interpreted as a small effect size, 0.31 (*r* = 0.30) as moderate, and 0.55 (*r* = 0.50) as large.

This study regarded Gates–MacGinitie Reading Test (GMRT), Lowa Tests of Basic Skills (ITBS), Group Reading Assessment and Diagnostic Evaluation (GRADE), Diagnostic Reading Analysis (DRA), Gray Oral Reading Test (GORT), Woodcock Reading Mastery Test (WRMT), Woodcock Language Proficiency Battery (WLPB), and Woodcock–Johnson Test of Achievement (WJ). Other measurements of reading comprehension were regarded as researcher-developed tests. This study regarded morpheme production, morpheme judgment as the standardized test for MA, phoneme deletion, and onset/rime/tone judgment as the standardized test for PA; all measurements for OS were regarded as the researcher-developed test.

To be conservative, the random-effects model was used to measure the effect size (Borenstein et al., 2009). This study also reported the 95% confidence interval (CI) and regarded those estimations as significant if CI did not include zero. A moderator analysis is performed for significant *Q* value if the analysis group contained more than four studies (Mol and Bus, 2011). If the moderator analysis performed insignificant *Q* value, that could be interpreted as the meta-correlation that did not interact significantly with other potential moderators.

To compare effect sizes, this study applied *Teta* as an indicator, where if |*Teta*| ≥ 1.96, the two effect sizes were regarded as significantly different. *Teta* was measured by following: *Teta* = *Diff* /*SE, Diff* = Fisher's *z*_1_ – Fisher's *z*_2_, *SE* = sqrt (Variance Fisher's *z*_1_ + Variance Fisher's *z*_2_).

This study reported publication bias through four ways: Rosenthal's fail-safe number, funnel plot, rank correlation test, and regression intercept test. Rosenthal's fail-safe number shows the number of missing studies with null effects that would make the previous correlations become insignificant (Borenstein et al., 2009). We used a trim-and-fill approach to show the funnel plot of adjusted effect sizes.

## Results

**Figure 1:**
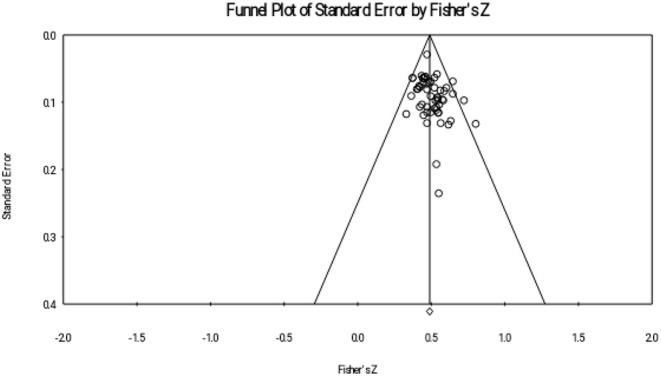
Funnel plot of morphological awareness (MA).

**Figure 2:**
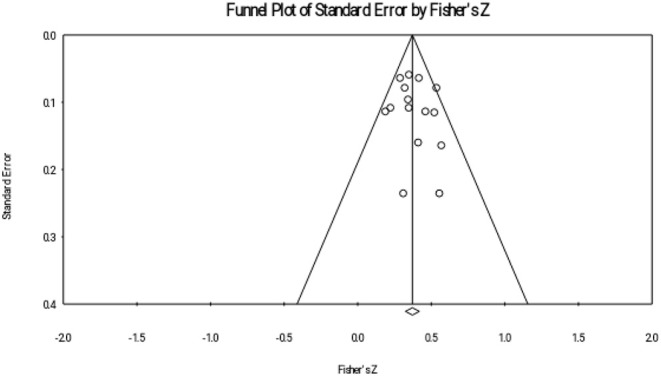
Funnel plot of orthographical skill (OS).

**Figure 3:**
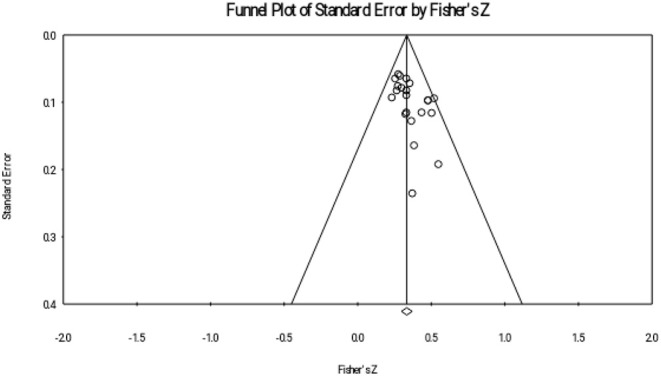
Funnel plot of phonological awareness (PA).

**Figure 4:**
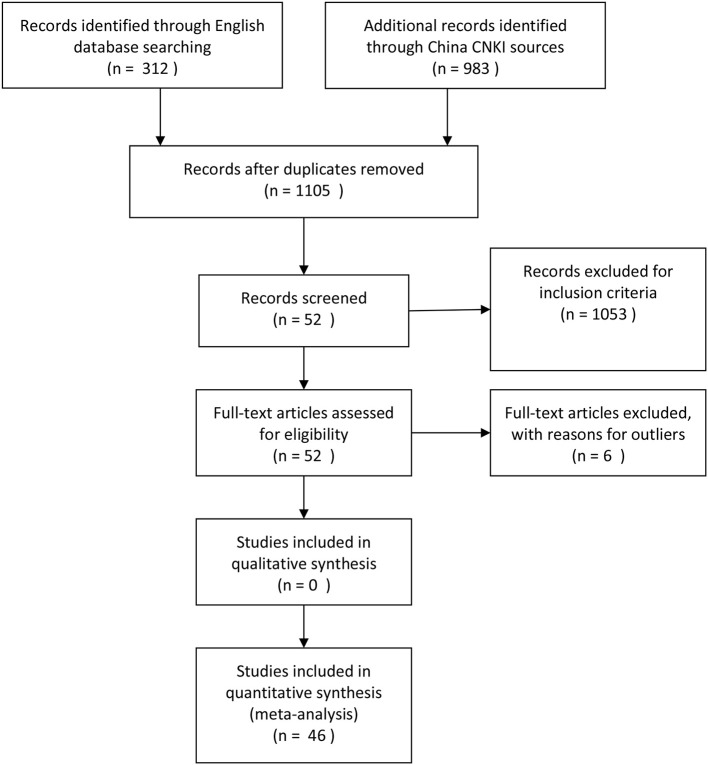
Flow chart.

The results of the meta-analyses are presented in three sections. Firstly, descriptive analysis was used (see Table 1). Secondly, interrelations between reading comprehension achievement and metalinguistic knowledge were explored in three subgroups (MA, PA, and OS). Thirdly, this study compared the effect sizes of the three metalinguistic factors. Finally, the publication bias examination were provided (see Figures 1–3). Materials search results and selection record were provided in Figure 4.

**Table 1:** Details for selected studies.

**Study no**.	**References**	**Area^a^**	**Material resources^b^**	**Sample size**
1	Zhang H., 2013	M	C	108
2	Lu and Zhang, 2015	M	C	108
3	Yan et al., 2007	M	C	118
4	Chang, 2010	M	C	175
5	Chen and Chen, 2008	M	C	30
6	Chen, 2011	M	C	287
7	Bai, 2002	M	C	61
7	Bai, 2002	M	C	60
8	Jia, 2015	M	C	40
9	Xiao and Ho, 2014	HK	E	90
10	Chang et al., 2014	M	C	78
11	Cheung et al., 2007	HK	E	88
11	Cheung et al., 2007	HK	E	88
12	Shu et al., 2006	M	E	75
12	Shu et al., 2006	M	E	77
13	Lin et al., 2015	M	C	115
14	Wu, 2011	M	C	78
14	Wu, 2011	M	C	78
15	Zhang et al., 2012	M	E	130
16	Yeung et al., 2013	HK	E	248
17	Ma and Lin, 2015	T	E	124
18	Guan et al., 2013	M	E	158
18	Guan et al., 2013	M	E	156
19	Zhang et al., 2014	M	E	245
19	Zhang et al., 2014	M	E	245
20	McBride-Chang et al., 2005	HK	E	100
20	McBride-Chang et al., 2005	M	E	100
21	Chen, 2015	T	C	164
22	Zhang and Yang, 2016	USA	E	21
23	Chung et al., 2011	HK	E	90
23	Chung et al., 2011	HK	E	30
24	Chik et al., 2012b	HK	E	83
24	Chik et al., 2012b	HK	E	119
24	Chik et al., 2012b	HK	E	59
25	Wang et al., 2006	USA	E	64
26	Tsung et al., 2017	M	E	111
26	Tsung et al., 2017	M	E	42
27	Chung et al., 2013	HK	E	78
28	Cheng et al., 2016	M	E	149
28	Cheng et al., 2016	M	E	127
29	Siu and Ho, 2015	HK	E	202
29	Siu and Ho, 2015	HK	E	202
29	Siu and Ho, 2015	HK	E	211
29	Siu and Ho, 2015	HK	E	211
30	Zhang and Yang, 2016	Singapore	E	108
30	Zhang and Yang, 2016	Singapore	E	108
31	Siu et al., 2016	HK	E	86
31	Siu et al., 2016	HK	E	168
31	Siu et al., 2016	HK	E	133
32	Leong et al., 2013	HK	E	1,164
33	Sun et al., 2013	M	E	80
34	Cheng et al., 2017	M	E	149
35	To et al., 2016	USA	E	61
35	To et al., 2016	USA	E	89
36	Bian, 2017	M	E	191
37	Wu et al., 2009	M	E	154
37	Wu et al., 2009	M	E	146
38	Zhang et al., 2012	M	E	130
39	Zhang H., 2013	M	E	245
40	Zhang et al., 2014	M	E	96
40	Zhang et al., 2014	M	E	96
41	Zhang and Yang, 2016	M	E	123
42	Leong and Ho, 2012	HK	E	73
43	Xia et al., 2017	M	E	21
44	Pan et al., 2016	M	E	294
45	Leong et al., 2011	HK	E	80
45	Leong et al., 2011	HK	E	80
46	Yeung et al., 2016	HK	E	239
46	Yeung et al., 2016	HK	E	239
47	Chen et al., 2018	M	E	164
48	Guan et al., 2014	M	E	261
48	Guan et al., 2014	M	E	242
48	Guan et al., 2014	M	E	246
49	Zhang et al., 2012	HK	E	164
49	Zhang et al., 2012	HK	E	164
50	Chik et al., 2012a	HK	E	272
51	Guan et al., 2014	M	E	261
51	Guan et al., 2014	M	E	242
51	Guan et al., 2014	M	E	246
52	Lam et al., 2012	Canada	E	46
52	Lam et al., 2012	Canada	E	34

*MA, morphological awareness; OS, orthographical skill; PA, phonological awareness*.

a *M, mainland; HK, Hong Kong; T, Taiwan*.

b *Material language: E, English; C, Chinese*.

c *L, grade 1 and grade 2 of primary school; M, grade 3 and grade 4 of primary school; H, grade 5 and grade 6 of primary school; S, secondary school; U, university*.

d *1, first language; 2, second language*.

### Overview of Studies

The majority (26) of the articles used a correlational design. Twenty-six studies reported the correlation between metalinguistic knowledge and English reading comprehension, and 47 studies reported the correlation between metalinguistic knowledge and Chinese reading comprehension. Sixteen studies reported the correlation between metalinguistic knowledge and reading comprehension in *L* grade group, 15 studies reported the correlation in *M* grade group, 24 studies reported the correlation in *H* grade group, 8 studies reported the correlation in *S* grade group, and 10 studies reported the correlation in *U* grade group. This study included all kinds of analytical methods in this review, aiming to investigate the relationships between variables. More specifically, six studies used a longitudinal design, and one article used an experimental design; 61 studies reported the correlation between MA and reading comprehension; 22 studies revealed the correlation between PA and reading comprehension; 15 studies revealed the correlation between OS and reading comprehension. All details can be found in Table 1.

**Table 1:** 

**Study no**.	**Grade group^c^**	**Language type^d^**	**MA**		**PA**		**OS**	
			**Fisher's *z***	***SE***	**Fisher's *z***	***SE***	**Fisher's *z***	***SE***
1	S	2	0.72	0.10	0.48	0.10		
2	S	2	0.58	0.10	0.48	0.10		
3	U	2			0.23	0.09		
4	H	2	0.42	0.08	0.27	0.08		
5	M	1			0.55	0.19		
6	M	1	0.57	0.06	0.16	0.06	0.35	0.06
7	U	2	0.57	0.13				
7	U	2	0.80	0.13				
8	U	2			0.38	0.16	0.57	0.16
9	H	1	0.42	0.11				
10	U	2					0.52	0.12
11	M	1					0.35	0.11
11	M	2					0.22	0.11
12	H	1	0.33	0.12	0.33	0.12		
12	H	1	0.55	0.12	0.50	0.12		
13	S	2	0.55	0.09	0.52	0.09		
14	M	2	0.47	0.12	0.44	0.12		
14	M	1	0.55	0.12	0.33	0.12		
15	U	2	0.27	0.09				
16	M	1	0.52	0.06				
17	U	2	0.37	0.09				
18	H	1	0.41	0.08				
18	H	1	0.40	0.08				
19	H	1	0.45	0.06			0.42	0.06
19	H	2	0.37	0.06			0.29	0.06
20	L	1	0.41	0.10	0.31	0.10		
20	L	1	0.42	0.10	0.33	0.10		
21	H	1	0.93	0.08				
22	U	2					0.31	0.24
23	S	1	0.47	0.11				
23	S	1	0.54	0.19				
24	H	1	0.54	0.11				
24	L	1	0.62	0.09				
24	H	1	0.63	0.13				
25	L	1	0.58	0.13	0.37	0.13		
26	H	2					0.34	0.10
26	H	2					0.41	0.16
27	S	1	0.50	0.12				
28	L	1			0.33	0.08		
28	L	1			0.33	0.09		
29	L	2	0.45	0.07				
29	L	1	0.48	0.07				
29	M	1	0.50	0.07				
29	M	2	0.65	0.07				
30	M	2	0.53	0.10				
30	M	2	0.55	0.10				
31	L	1	0.52	0.11				
31	H	1	0.42	0.08				
31	M	1	0.65	0.09				
32	S	1	0.47	0.03				
33	L	1			0.60	0.11		
34	L	1	0.56	0.08	0.27	0.08		
35	U	2	0.47	0.13				
35	U	2	0.54	0.11				
36	U	2	0.47	0.07				
37	L	1	0.47	0.08				
37	L	1	0.59	0.08				
38	U	2	0.25	0.09				
39	H	2	0.37	0.06				
40	H	2	0.56	0.10				
40	H	1	0.44	0.10				
41	L	1	0.50	0.09				
42	S	1	0.45	0.12				
43	H	1	0.55	0.24	0.37	0.24	0.56	0.24
44	H	1	0.54	0.06	0.28	0.06		
45	H	1					0.46	0.11
45	H	2					0.19	0.11
46	L	1			0.33	0.07		
46	M	1			0.26	0.07		
47	H	1	0.83	0.08				
48	H	1	0.46	0.06				
48	H	1	0.45	0.06				
48	M	1	0.45	0.06				
49	L	1	0.52	0.08	0.30	0.08	0.54	0.08
49	L	1	0.60	0.08	0.54	0.08	0.32	0.08
50	L	1	0.44	0.06	0.29	0.06		
51	H	1	0.46	0.06				
51	H	1	0.45	0.06				
51	M	1	0.45	0.06				
52	L	2	0.40	0.15	0.51	0.15	0.51	0.15
52	L	2	0.97	0.18	0.63	0.18	0.63	0.18

### Meta-Analysis

Table 2 demonstrates that the correlation between OS and reading comprehension was moderate (Fisher's *z* = 0.37), and no selected moderators had any significant interactive effect (*Q* = 16.21, *p* > 0.05) on this correlation. The correlation between PA and reading comprehension was moderate (Fisher's *z* = 0.33), and no significant moderators that were selected in the current study had any significant interaction (*Q* = 18.35, *p* > 0.05) with this correlation. The correlation between MA and reading comprehension achievement was large (Fisher's *z* = 0.49), and no selected moderator had any significant interaction effect (*Q* = 50.99, *p* > 0.05) on this correlation.

**Table 2:** Metalinguistic knowledge with reading comprehension achievement.

**Variables**	***k***	**Sample *N***	**Fisher's *z***	***V***	**95%CI**	***Q***	***I*^**2**^**	***N* fail-safe**	***Teta***
OS	15	1,754	0.37	0.001	[0.32,0.42]	16.12	13.12	97	MA > PA;
MA	57	9,544	0.49	<0.001	[0.47,0.51]	50.99	<0.001	501	MA > OS;
PA	22	2,916	0.33	<0.001	[0.30,0.37]	18.35	<0.001	125	OS = PA

*V, variance; MA, morphological awareness; PA, phonological awareness; OS, orthographical awareness*.

Regarding correlation comparison, the difference between OS and PA was not significant (*Teta* < 1.96), yet the difference between OS and MA was considerable (*Teta* = 3.08, *p* < 0.01). The difference between MA and PA was significant (*Teta* = 9.90, *p* < 0.001).

## Discussion

The current empirical review had two main findings. Firstly, for Chinese primary, secondary, and university students, the correlation between each category of metalinguistic knowledge (MA, OS, or PA) and reading comprehension was independent and was not moderated significantly by selected moderators, implicating that reading stages' effect was not significant between metalinguistic knowledge and reading comprehension in Chinese students. Secondly, for Chinese students, the MA had a significantly higher correlation with reading comprehension than PA and OS in both Chinese scripts text reading and English scripts text reading.

### Morphological Awareness Effect in Chinese Students

The current meta-analytic review presents a different conclusion in Castles et al. (2018) opinion, which claimed that the PA played the most important role on word identification and cognition process in English scripts' reading comprehension for those students who regarded the alphabetical language as the first language; that is, that for Chinese students, the MA had a larger effect size than PA in both Chinese and English scripts' reading comprehension. This reinforces the argument put forward by several researchers that MA is fundamental to word reading in Chinese participants (e.g., Song et al., 2016; Ruan et al., 2018). The reason should be the morpho-syllabic nature of the Chinese writing system (Cheng et al., 2016), with a great number of Chinese words with meanings that can be inferred from their morphemes. This means that even though the readers may not know how to pronounce the morphemes accurately, they can guess the character's semantic meaning from its morpheme. For example, Zhang (2017) found that MA enhanced Chinese learners to understand the unfamiliar words through intro-word morphological properties during textual reading. Chung and Leung (2008) reported a very low percentage (~25%) of Chinese characters with a regular phonetic radical, in which readers did not process effectively the target character through the phonetic radical; the importance of PA in Chinese students might downgrade. The second reason that MA had a larger correlation with reading comprehension achievement is the training system of Chinese students. For example, young Chinese students, without any phonetic transcription at the beginning, were suggested to focus on holistic character reading (Tong et al., 2009). Students developed graph MA on text comprehension because Chinese semantic radicals encode target characters' meaning. The learning strategy would transfer to L2 learning. Even though English reading comprehension consists of a series of alphabets, most students still apply the learning strategy of Chinese when they learn English. They recognize English words by guessing the meaning of each morpheme, such as the affix “re-” represented the semantic meaning “repeated.” When readers processed the new words from the text, they would first try to find the stem of the target word and then guess its meaning.

### Phonological Awareness and Orthographical Skill Effect in Chinese Students

Both PA and OS had a medium correlation with reading comprehension in Chinese students' text reading comprehension performance. Firstly, the significant medium correlation between PA and reading comprehension was consistent with Song et al. (2016) study, suggesting that the phonetic radical of characters provided some clues for readers to process the characters' semantic meaning. The moderate correlation effect size between PA and reading comprehension for Chinese students may come from the first language background; that is, the percentage of Chinese characters' pronunciation matching phonetic radical was very low (Chung and Leung, 2008); students did not use PA on word semantic meaning process as frequently as MA, resulting in the smaller correlation between PA and reading comprehension than MA in Chinese students. Secondly, PA and OS had similar correlation effect size with reading comprehension. This finding was consistent with Koda's (2005) hypothesis, which posited that the OS was a complex system with a basic knowledge of the sound–symbol relationship. Previous studies confirmed that OS has a close relationship with PA because researchers have reported that readers' OS development relies on their PA development (e.g., Ehri, 1992). For example, the letter–sound spelling rule of PA was considered to help students write words by retrieving spellings' form (phonemic segmentation skill) from memory (Griffith, 1991); that is, that PA helps readers to develop their knowledge of the target language characters' position. Similar results also confirmed the very close relationship between OS and PA.

### Reading Comprehension and Metalinguistic Knowledge

The current study provided evidence that MA, PA, and OS showed an independent correlation with reading comprehension achievement, which did not interact with any significant moderators. The effect of the reading stage was not been significantly found in the current study. The result of the MA correlation was large and not moderated by any language type in Chinese participants, regardless whether in Chinese or English scripts' reading comprehension. The major reason should be the requirement of the reading task. Participants were required to do a silent reading task, in which MA played a major role during the cognition progress through a single-character or single-word semantic meaning inference. Another reason is due to the participants' cognitive styles of understanding logographic Chinese, which is based on MA that is taught by their teachers. Therefore, Chinese participants may still tend to use MA to do comprehension tasks in both Chinese and English scripts, regardless in which reading stages. This result supported the Seidenberg (2011) statement of same set of mapping rules between speaking and writing on word semantic meaning identification for Chinese readers. That is the basic graphic unit of the character, which represents a morpheme (not a phoneme) that determined Chinese character cognition process. An alternative reason should be richness of homophones in Chinese characters (Kuo and Anderson, 2006; Chow et al., 2008); students did not distinguish and identify words with the same pronunciation by simply relying on PA, which means that the spelling-to-sound mappings was not applicable for Chinese reading comprehension. In contrast, most Chinese characters' semantic meaning was identified from the regular and informative morphemes; therefore, Chinese readers tend to rely more on MA on target character or word cognition (Tong et al., 2011; Ruan et al., 2018).

The current result demonstrated that the PA was not checked by any significant moderators. This finding was different from the findings by Mayberry et al. (2011) and Swanson et al. (2003), who showed that grade group moderated the correlation between PA and reading comprehension achievement. The reason may be due to the linguistic characteristics of the target text, which were available in one alphabetical script (spelling-to-sound mappings); in particular, little PA could be used in Chinese scripts' text comprehension task. Because of this, the transfer effect of MA may occur when they switch from Chinese reading comprehension to English reading comprehension. That is to say, the amount of PA knowledge (i.e., grammar and component combination rules), which could apply to Chinese students' reading comprehension performance, is limited. The independent effect of metalinguistic knowledge on reading comprehension tasks for Chinese students, informing the function of metalinguistic knowledge on text comprehension, was word identification and word recognition only for text comprehension process; the aim of different reading stages did not impact the contribution of metalinguistic knowledge on reading comprehension.

### Limitations and Implications

The current study has several limitations. Firstly, the participants are typically developing school students, whereas those who had special education needs (e.g., deaf) were not reported. Secondly, this study only included studies focusing on English and Chinese reading comprehension for Chinese students. Thus, studies of reading comprehension in other target languages were not reported. The moderator analysis showed that the correlation between metalinguistic knowledge and reading comprehension was not significantly moderated by other comprehension factors, indicating that in any grade group or language type (Chinese or English scripts' reading), reading comprehension ability could be predicted by metalinguistic knowledge. Secondly, since MA played the most important role in reading comprehension of the three metalinguistic knowledge, future intervention studies should pay more attention to MA training to improve Chinese students' reading comprehension performance.

## Conclusion

Based on the combined results of 73 independent studies conducted on 10,793 students, this meta-analysis has shown that the correlation between the three types of metalinguistic knowledge and reading comprehension was not moderated significantly by moderators. MA has a larger correlational effect than PA and OS on reading comprehension in Chinese students. PA and OS shared similar effects on reading comprehension achievement in Chinese students. The results highlighted the different effects of metalinguistic knowledge in Chinese and English reading comprehension among Chinese students in all grade groups. In addition, given the fact that grade group did not have any significant moderating effect, implicating reading stages did not impact the function of metalinguistic knowledge on text reading comprehension for Chinese students' Chinese or English scripts' reading.

## Data Availability Statement

The datasets generated for this study are available on request to the corresponding author.

## Author Contributions

YD draft almost the manuscript. S-NP did coding and proof reading of the manuscript. Y-KS did data collection, coding, and data analysis. SW did data analysis for result confirmation. W-SW provided the comments to draft and revised the draft.

### Conflict of Interest

The authors declare that the research was conducted in the absence of any commercial or financial relationships that could be construed as a potential conflict of interest. The reviewer (SSY) declared a shared affiliation, with no collaboration, with several of the authors, (SW) and (YS), to the handling editor at time of review.
